# Unravelling the potential of social prescribing in individual-level type 2 diabetes prevention: a mixed-methods realist evaluation

**DOI:** 10.1186/s12916-023-02796-9

**Published:** 2023-03-13

**Authors:** Sara Calderón-Larrañaga, Trish Greenhalgh, Megan Clinch, John Robson, Isabel Dostal, Fabiola Eto, Sarah Finer

**Affiliations:** 1 Centre for Primary Care, Wolfson Institute of Population Health, Queen Mary University of London, Yvonne Carter Building, 58 Turner Street, London, E1 2AB UK; 2 Bromley By Bow Health Partnership, XX Place Health Centre, Mile End Hospital, Bancroft Rd, Bethnal Green, London, E1 4DG UK; 3 Nuffield Department of Primary Care Health Sciences, University of Oxford, Radcliffe Primary Care Building, Radcliffe Observatory Quarter, Woodstock Rd, Oxford, OX2 6GG UK; 4 Barts Health NHS Trust, Newham University Hospital, Glen Rd, London, E13 8SL UK

**Keywords:** Social prescribing, Diabetes prevention, Health promotion, Primary care

## Abstract

**Background:**

Social prescribing (SP) usually involves linking patients in primary care with services provided by the voluntary and community sector. Preliminary evidence suggests that SP may offer a means of connecting patients with community-based health promotion activities, potentially contributing to the prevention of long-term conditions, such as type 2 diabetes (T2D).

**Methods:**

Using mixed-methods realist evaluation, we explored the possible contribution of SP to individual-level prevention of T2D in a multi-ethnic, socio-economically deprived population in London, UK. We made comparisons with an existing prevention programme (NHS Diabetes Prevention Programme (NDPP)) where relevant and possible. Anonymised primary care electronic health record data of 447,360 people 18+ with an active GP registration between December 2016 and February 2022 were analysed using quantitative methods. Qualitative data (interviews with 11 primary care clinicians, 11 social prescribers, 13 community organisations and 8 SP users at high risk of T2D; 36 hours of ethnographic observations of SP and NDPP sessions; and relevant documents) were analysed thematically. Data were integrated using visual means and realist methods.

**Results:**

People at high risk of T2D were four times more likely to be referred into SP than the eligible general population (RR 4.31 (95% CI 4.17–4.46)), with adjustment for socio-demographic variables resulting in attenuation (RR 1.33 (95% CI 1.27–1.39)). More people at risk of T2D were referred to SP than to NDPP, which could be explained by the broad referral criteria for SP and highly supportive (proactive, welcoming) environments. Holistic and sustained SP allowed acknowledgement of patients’ wider socio-economic constraints and provision of long-term personalised care. The fact that SP was embedded within the local community and primary care infrastructure facilitated the timely exchange of information and cross-referrals across providers, resulting in enhanced service responsiveness.

**Conclusions:**

Our study suggests that SP may offer an opportunity for individual-level T2D prevention to shift away from standardised, targeted and short-term strategies to approaches that are increasingly personalised, inclusive and long-term. Primary care-based SP seems most ideally placed to deliver such approaches where practitioners, providers and commissioners work collectively to achieve holistic, accessible, sustained and integrated services.

**Supplementary Information:**

The online version contains supplementary material available at 10.1186/s12916-023-02796-9.

## Background

Social prescribing (SP) usually involves linking patients in primary care with services provided by the voluntary and community sector (VCS) [1]. Activities are typically non-medicalised, provided locally and have a wide remit (from lifestyle programmes to welfare advice and/or community engagement initiatives, depending on patients’ needs and local availability) [2, 3]. In the UK, the link between primary care and the VCS is facilitated by a link worker (also referred to as a social prescriber), whose role ranges from signposting to patients’ needs assessment, ongoing support or the development of new VCS activities where gaps exist [4]. As part of the NHS Long-Term Plan, NHS England set to recruiting enough link workers to make the service available in every GP practice by 2023/2024 [5, 6].

SP is expected to advance the prevention and management of long-term conditions, such as type 2 diabetes (T2D) [7, 8], by encouraging a healthier lifestyle, self-management and personalised care [9–11]. However, the current evidence base for SP and its role in specific areas of health need, such as T2D, is scarce. This study focused on T2D prevention and investigated the role of primary care-based SP in people at high risk of the condition based on the following considerations. First, T2D is a major public health concern; it is common (and increasingly so) and is associated with reduced quality of life, life expectancy and considerable socio-economic consequences [12–14]. Both individual behavioural risk factors and socio-economic determinants appear to be major driving forces behind escalating T2D epidemics and health inequalities [15, 16]. Second, many community-based activities accessed through SP focus on healthy lifestyle, including weight management, dietary recommendations and physical activity [1, 17–19], which also underpin existing T2D prevention behavioural programmes, such as the NHS Diabetes Prevention Programme (NDPP) [20]. However, by also acknowledging people’s underlying social constraints, SP may offer a means of providing contextually sensitive and holistic health promotion [21], in line with best practice recommendations for individual-level T2D prevention [22]. Third, NDPP focuses purely on the promotion of behaviour change in patients at high risk of developing diabetes. Delivered at scale through (mostly private) independent providers, NDPP has shown low uptake and high attrition rates, especially amongst socio-economically deprived and diverse ethnic groups [23–25]. There is thus a need for T2D prevention strategies to increase attention to patients’ wider social context and improve their reach to those in greatest need.

This study aimed to investigate the possible role of SP in T2D prevention and evaluate the extent to which it may complement and inform existing preventative approaches (NDPP). We hypothesised that the reach and equity of access of SP and NDPP across high-risk patients within a multi-ethnic, socio-economically deprived population could differ and sought to understand why (and how) possible differences could occur. Using a realist mixed-methods design, we investigated, first, whether (and the extent to which) SP might reach high-risk patients in greatest health and social need; second, what “good” practice in SP relevant to people at high risk of T2D might look like; and third, key ingredients of SP that might contribute (or not) to T2D prevention.

## Methods

**Fig. 1:**
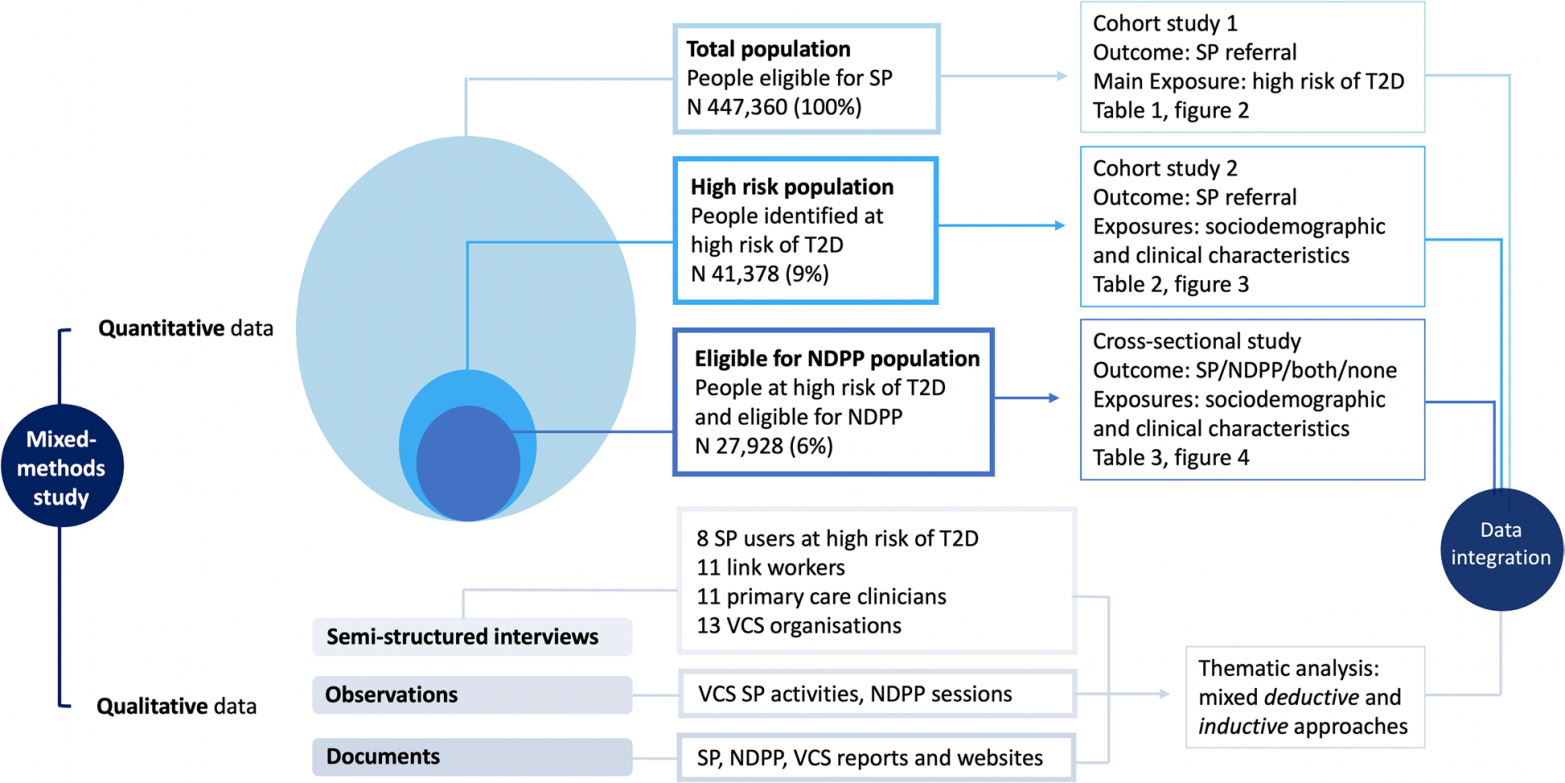
Overview of study data sources and analysis. Legend: SP Social Prescribing; NDPP NHS Diabetes Prevention Programme; T2D Type 2 Diabetes; VCS Voluntary and Community Sector

Quantitative and qualitative data were collected and analysed concurrently between November 2020 and March 2022 (see Fig. 1 for an overview of data sources and analysis) [26]. We followed RAMESES reporting standards for realist evaluations [27].

### Theoretical approach: realist evaluation

This study used mixed-methods realist evaluation [28], informed by a previous realist synthesis undertaken by this team [21]. Realist evaluation is a theory-driven methodology that seeks to facilitate a deep understanding of how complex interventions, such as SP, work and in what circumstances [29]. Causal narratives are central to realist evaluation but have a wider conception that goes beyond the criterion of observability to also account for the non-physical and unobservable—for example, the conscious or unconscious reasoning which drives individuals’ decisions and actions [30]. In order to explore these underlying causal explanations, realist methodology is designed to tease out what are known as context-mechanism-outcome configurations (CMOCs) [31]. A CMOC is a hypothesis that the programme works (or does not work) (O) because of the action of some underlying mechanisms (M), which come into operation only in particular contexts (C) [32]. These causal explanations (also referred to as programme theories [33]) are made explicit and tested, confronted and refined iteratively throughout the research using a range of empirical data [27].

Realist evaluations have been successfully used to illuminate the resources (mechanisms within specific contexts) which influence the nature and potential impact of complex interventions, such as SP [34, 35]. This study builds on existing published literature using realist methodology to provide a novel evaluation of the potential role of SP in a specific context of preventative health (relating to T2D). Realist evaluation also provides a useful framework for mixed-methods research, whereby quantitative data may help identify patterns of SP practice that are then explored further using rich qualitative insights from SP users and service providers (or vice versa) [36].

### Study setting and intervention

The study was based in Tower Hamlets, a multi-ethnic inner-city borough in east London, UK, with an estimated population of 310,300. Tower Hamlets is one of the most deprived boroughs in the UK, characterised by its great ethnic diversity. Overall and T2D-related health outcomes are significantly poorer than the national average despite high-quality primary and secondary care [37–39].

Tower Hamlets has been at the forefront of SP implementation and delivery and has a long-established local VCS. Since 2016, all patients registered with a Tower Hamlets GP practice, aged over 18 and expressing a “non-clinical” support need are eligible for the local SP programme [18]. In line with NHS requirements for SP roll-out and delivery, people can self-refer or be referred by any local primary care professional to their named link worker, who is usually located in the GP practice with full access to patients’ medical records [11]. Based on the identified needs, link workers may offer signposting or referral to relevant VCS resources, and/or further face-to-face or telephone follow-up appointments. Most VCS activities accessed through SP were related to lifestyle (such as exercise, healthy eating and weight management) or welfare advice (such as debt, benefits, housing issues), while the duration of activities and support varied across different services and organisations [18]. During the early months of the COVID-19 pandemic, the service had to adapt its working practices to factors such as remote-by-default policies for GP access, working from home and local agencies providing limited or restricted services. All link workers were provided with remote access to medical records and homeworking equipment to ensure service continuation and the provision of phone and/or video appointments to referred patients.

Tower Hamlets was also one of the 27 areas across the country chosen to be part of the first wave roll-out of NDPP in 2016. The programme targets individuals at high risk of T2D, demonstrated by a diagnosis of non-diabetic hyperglycaemia or previous gestational diabetes. It is delivered by a private provider and commissioned by NHS England, with referral volume used as the main metric of site activity. General practice involvement is limited to the identification and referral of patients, enhanced through various incentives and support strategies. The core NDPP intervention consists of group-based sessions offering behavioural change content intended to achieve improvements in diet, physical activity levels and weight. The course consists of a minimum of 16 h of contact time over at least 9 months [20]. During the pandemic, sessions were held online or over the phone, depending on patients’ preference.

### Quantitative data

The study population was all adults (18+ years) without diabetes, registered with a GP in any of 35 practices in Tower Hamlets, east London, between 1 December 2016 and 14 February 2022. Electronic health record data were accessed from these GP practices via the Clinical Effectiveness Group, Queen Mary University of London, and pseudo anonymised at source under NHS and local information governance and data security policies. Detailed inclusion and exclusion criteria and study variables are presented in Additional file 1: Table S1.

We undertook a cohort study [1] of people eligible for SP (18+ and with an active GP registration in Tower Hamlets) to investigate the independent association between being at high risk of T2D and being referred into SP. This population was selected using the latest date of GP registration or the study period start date. In a second cohort study [2], we investigated the clinical and socio-demographic features associated with being referred into SP in a population at high risk of T2D. In this study, high risk of T2D was determined as history of gestational diabetes, Q Diabetes risk calculator score (QDiabetes®-2018 [40]) equal or above to 20, fasting blood glucose 5.5–6.9 mmol/L, HBA1c 42–47 mmol/mol, diagnosis of non-diabetic hyperglycaemia and/or pre-diabetes or history of referral into NDPP. We selected this population as all those in a T2D high-risk state, using the latest date of diagnosis, date of GP registration or study period start date. We excluded patients with diabetes if they had been diagnosed prior to the end of follow-up. People in both cohort studies were censored at the earliest date of the coded referral into SP (primary outcome), death, deregistration with the practice or the study end date. Finally, we undertook a cross-sectional study to investigate the clinical and socio-demographic features associated with a coded referral into SP, NDPP, both or neither amongst people eligible for NDPP. The exposures and outcomes for each of these studies were described using percentages (categorical variables) or median and IQR (continuous variables). Poisson fixed effect models were used to calculate all rate and rate ratios (RR) (95% CI) in cohort studies 1 and 2. Fixed effect multinomial regression models were used to calculate all odds and odd ratios (OR) (95% CI) comparing referral into SP with referral into NDPP (baseline category) in the cross-sectional study [3]. Potential confounders were included in the multivariate models to calculate the independent association of the different risk factors with being referred to SP and/or NDPP. Models were constructed based on the hierarchical relationship of variables established in a conceptual framework [41], previous assumptions on causality (determined using a direct acyclic graph (DAG) [42]) and assessment of the correlation between independent variables (collinearity). All analyses were conducted using Stata (version 17).

### Qualitative data

We collected different types of qualitative data to evaluate the actual delivery of SP, including key ingredients that could explain its potential in reaching high-risk patients with greatest health and social need. Qualitative data sources and sample characteristics are shown in detail in Additional file 2: Tables S1-S5. Briefly, we drew on [1] semi-structured interviews with 8 SP users at high risk of T2D, 11 primary care clinicians (GPs, nurses, a physiotherapist and health care assistant), 11 link workers and 13 local VCS organisations accessed through SP; [2] 36 hours of ethnographic observations of community-based SP activities (including holistic weight management and physical activity programmes), privately delivered NDPP sessions and VCS meetings; and [3] documents about the local SP scheme, NDPP programme and VCS organisations (including descriptions of activities on offer, target population, eligibility criteria and evaluation reports). Interviews and observations were initially conducted remotely (online or over the phone) and later shifted to face to face in compliance with COVID-19 safety measures. The sample size was determined iteratively guided by data saturation. Interview transcripts, fieldnotes and documents were initially analysed thematically. We combined a broadly deductive analytic approach (to test and refine previous realist review findings) with a more inductive analysis (to explore new, unexpected findings related to the specific intervention and local contexts) [43]. Data were analysed in six iterative stages (repeated reading, development of initial codes, generation, review and naming of themes, and writing), guided by the framework proposed by Braun and Clarke [44]. We sought negative cases (including contradictory findings and inconsistencies across and within data) and triangulated within the research team to enhance validity and rigour [45]. Data management was supported by NVivo V.10 software.

### Integration of quantitative and qualitative findings

Data were integrated using visual means (also referred to as *joint display integration* [26]), which enabled drawing new insights beyond the information gained from the separate quantitative and qualitative results [46]. Referral patterns and practices were further contextualised in light of rich qualitative data to develop explanations of how and why they occurred and their meaning for people at high risk of T2D. At this point, a realist logic of analysis was applied, which involved making inferences about whether different components of the data were functioning primarily as context, mechanism or outcome (and the relationships between them) [32]. CMOCs were further reviewed, refined and tested against empirical data, our previous realist review [21] and through discussion with the research team and stakeholders.

## Results

The extent to which SP succeeded in reaching high-risk people (quantitative findings) and the mechanisms by (and contexts in) which this was achieved (qualitative findings) are explained below.

### Referral and non-referral to social prescribing in a population at high risk of type 2 diabetes: quantitative findings

**Fig. 2:**
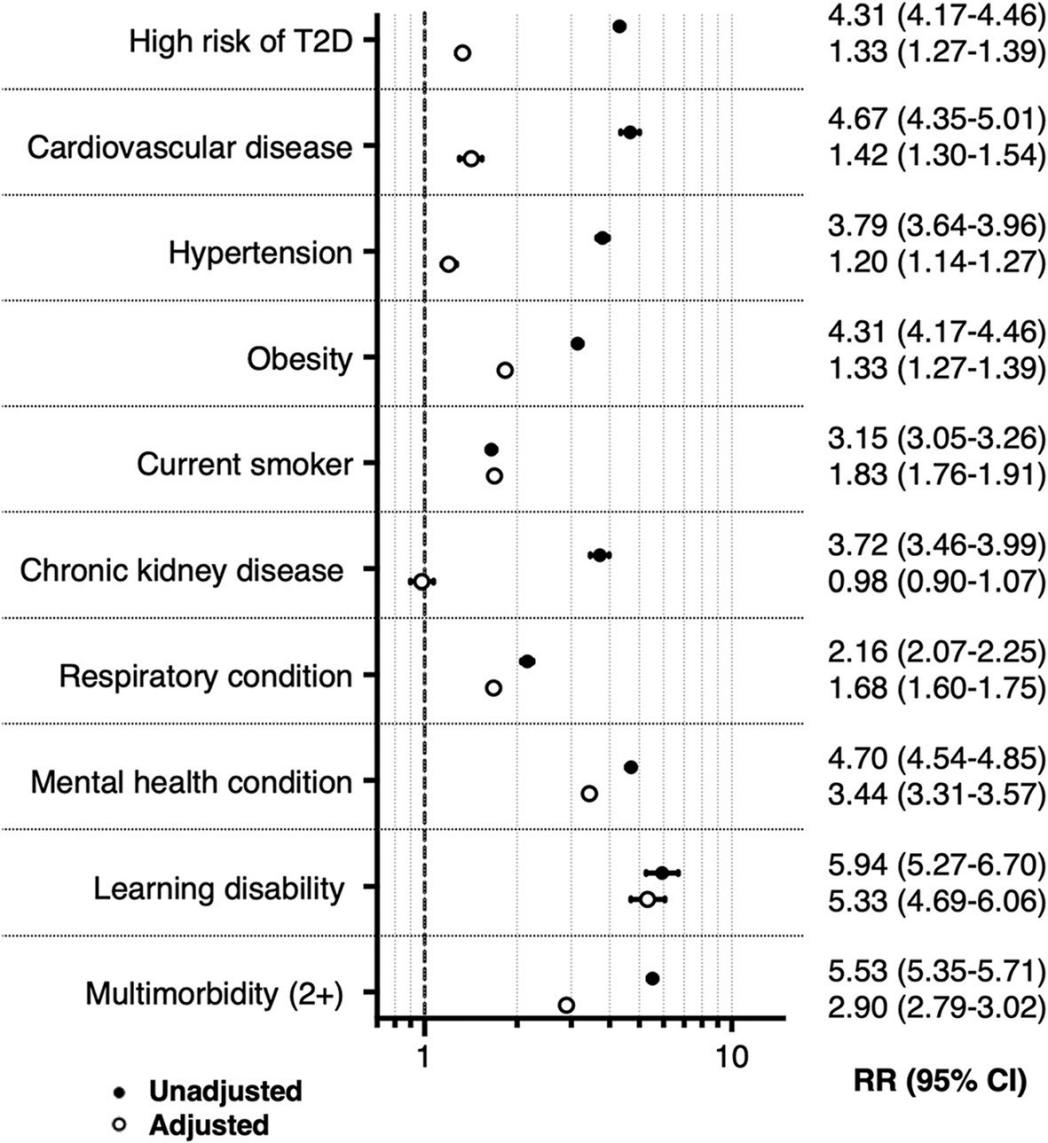
Distribution of clinical features and their association with referral into SP among the total study population, cohort study 1. Legend: T2D Type 2 Diabetes. Cardiovascular disease includes Ischemic Heart Disease, Peripheral Arterial Disease, and/or Stroke and Transient Ischemic Attack. Respiratory condition includes Asthma and/or COPD. See Additional File 1: Table S2 for information on the variables included in each adjusted model

A total of 447,360 people eligible for SP were enrolled between December 2016 and February 2022. Over a median follow-up period of 4.5 years, 15,450 referrals into SP were observed (1,604,194 person-years). As shown in Table 1, people referred into SP were more likely to be female (RR 1.74 (95% CI 1.68–1.80)), socio-economically deprived (RR 2.18 (95% CI 1.97–2.40)), black (RR 2.02 (95% CI 1.90–2.15)), South Asian (RR 2.27 (95% CI 2.18–2.36)) or Arab (RR 2.54 (95% CI 2.07–3.13)) than the general population eligible for the service. Those at high risk of T2D were four times more likely to be referred to SP (RR 4.31 (95% CI 4.17–4.46)), with adjustment for socio-demographic variables attenuating the association (RR 1.33 (95% CI 1.27–1.39)). Similarly, people living with long-term conditions (including cardiovascular diseases (RR 4.67 (95% CI 4.35–5.01)), obesity (RR 3.15 (95% CI 3.05–3.26)), mental health conditions (RR 4.70 (95% CI 4.54–4.85))) and multimorbidity (RR 5.53 (95% CI 5.35–5.71)) were at higher risk of SP referral, which also attenuated with adjustment for socio-demographic variables (see Fig. 2 and Additional file 1: Table S2).

**Table 1:** Distribution of socio-demographic characteristics within the total study population and their association with referral into SP, cohort study 1

Variables		Total *N* 447,360 (%)	Events (SP) *N* 15,454 (%)	Rates per 1000 P/Y	RR (95% CI)	*P* value	RR^a^ (95% CI)	*P* value
Gender	Male	219,795 (49.1)	5990 (38.8)	7.5 (7.3–7.7)	1	<0.001	1	<0.001
	Female	227,498 (50.9)	9462 (61.2)	11.8 (11.5–12.0)	1.57 (1.52–1.62)		1.74 (1.68–1.80)	
	Missing	67 (0.01)	2 (0.01)					
Age	Median (IQR)	33 (28, 42)	43 (33, 57)					
Ethnicity	White	184,631 (41.3)	5031 (32.6)	7.5 (7.3–7.7)	1		1	
	South Asian	93,722 (21.0)	5513 (35.7)	15.5 (15.1–15.9)	2.07 (1.99–2.15)	<0.001	2.27 (2.18–2.36)	<0.001
	Chinese	16,191 (3.6)	77 (0.5)	1.4 (1.1–1.8)	0.19 (0.15–0.24)	<0.001	0.25 (0.20–0.32)	<0.001
	Black	18,298 (4.1)	1290 (8.4)	19.1 (18.1–20.2)	2.55 (2.40–2.72)	<0.001	2.02 (1.90–2.15)	<0.001
	Arab	1931 (0.4)	91 (0.6)	16.6 (13.6–20.4)	2.22 (1.81–2.73)	<0.001	2.54 (2.07–3.13)	<0.001
	Mixed/others	23,491 (5.3)	694 (4.5)	9.1 (8.5–9.9)	1.22 (1.23–1.32)	<0.001	1.37 (1.26–1.48)	<0.001
	Missing	109,096 (24.4)	2758 (17.9)					
IMD quintiles	1st and 2nd (most deprived)	371,964 (83.2)	14,167 (91.7)	10.6 (10.4–10.8)	2.13 (1.98–2.28)	<0.001	2.18 (1.97–2.40)	
	4th and 5th (least deprived)	31,064 (6.9)	462 (3.0)	4.1 (3.6–4.5)	1		1	
	Missing	3372 (0.8)	15 (0.1)					

*IMD* Index of Multiple Deprivation, *IQR* interquartile range

^a^Adjusted by the remaining socio-demographic variables (gender, ethnicity, IMD) and time variables (age, year)

**Fig. 3:**
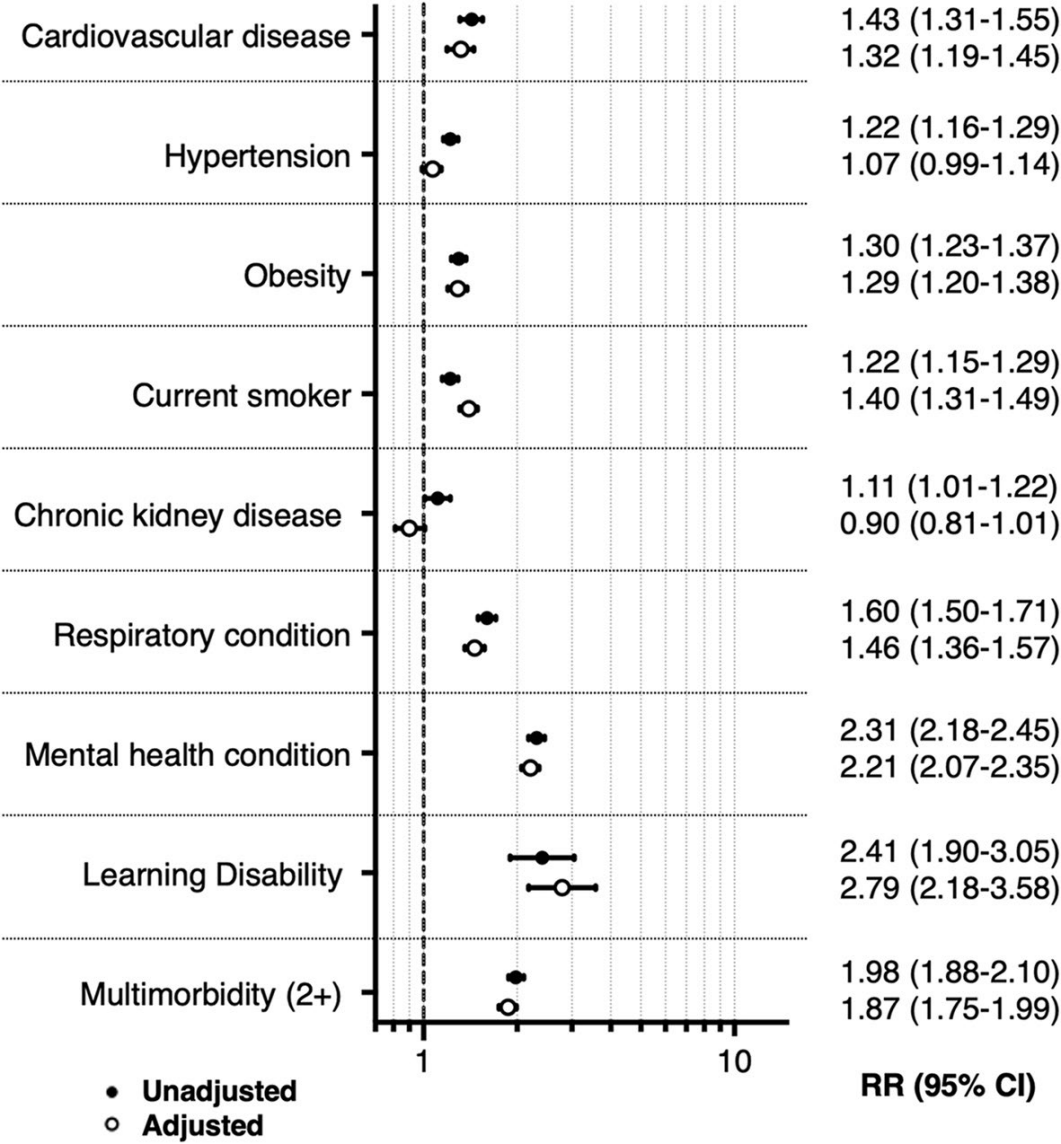
Distribution of clinical features and their association with referral into SP among people at high risk of T2D, cohort study 2. Legend: See Additional File 1: Table S3 for information on the variables included in each adjusted model

Of the total population eligible for SP, 9.2% (41,378) were identified as high risk of T2D and were, therefore, included in cohort study 2. The median follow-up period for these high T2D risk individuals was 5.2 years, which resulted in 5226 referrals into SP (164,614 person-years). The pattern of findings for high-risk patients was similar to the general population. As shown in Table 2, those referred into SP were more likely to be female (RR 1.54 (95% CI 1.46–1.64)), socio-economically deprived (RR 1.83 (95% CI 1.53–2.19)) and South Asian (RR 1.16 (95% CI 1.08–1.25)). Similarly, living with cardiovascular disease (RR 1.43 (95% CI 1.31–1.55)), obesity (RR 1.30 (95% CI 1.23–1.37)), mental health conditions (RR 2.31 (95% CI 2.18–2.45)) or multimorbidity (RR 1.98 (95% CI 1.88–2.10)) significantly increased the risk of being referred into SP. Adjustment for confounders, however, resulted in a lower attenuation, suggesting greater homogeneity in terms of socio-demographic features across this high-risk sample (see Fig. 3 and Additional file 1: Table S3). In all cases, the referral rate was higher at older ages and during the first year of service roll-out (December 2016–December 2017) (Additional file 1: Tables S4 and S5).

**Table 2:** Distribution of socio-demographic characteristics within the study population at high risk of T2D and their association with referral into SP, cohort study 2

Variables		Total *N* 41,378 (%)	Events (SP) *N* 5226 (%)	Rates per 1000 P/Y	RR (95% CI)	*P* value	RR^a^ (95% CI)	*P* value
Gender	Male	20,419 (49.4)	2127 (40.7)	25.5 (24.5–26.7)	1	<0.001	1	<0.001
	Female	20,957 (50.8)	3099 (59.3)	38.1 (36.8–39.5)	1.49 (1.41–1.58)		1.54 (1.46–1.64)	
	Missing	2 (0.0)	0 (0.0)					
Age	Median (IQR)	52 (43, 64)	55 (45, 66)					
Ethnicity	White	11,253 (27.2)	1494 (28.6)	32.0 (30.4–33.6)	1		1	
	South Asian	19,406 (46.9)	2469 (47.2)	32.7 (31.4–34.0)	1.02 (0.96–1.09)	0.489	1.16 (1.08–1.25)	<0.001
	Chinese	533 (1.3)	23 (0.4)	10.6 (7.1–16.0)	0.33 (0.22–0.50)	<0.001	0.36 (0.24–0.64)	<0.001
	Black	3275 (7.9)	469 (9.0)	35.1 (32.0–38.4)	1.10 (0.99–1.22)	0.080	1.10 (0.99–1.23)	0.069
	Arab	142 (0.3)	24 (0.5)	43.8 (29.3–65.3)	1.37 (0.92–2.05)	0.126	1.49 (0.99–2.24)	0.053
	Mixed/others	1352 (3.3)	177 (3.4)	33.9 (29.3–39.3)	1.06 (0.91–1.24)	0.460	1.11 (0.95–1.30)	0.178
	Missing	5417 (13.1)	570 (10.9)					
IMD quintiles	1st and 2nd (most deprived)	37,283 (90.1)	4878 (93.3)	32.9 (32.0–33.9)	1.90 (1.59–2.27)	<0.001	1.83 (1.53–2.19)	<0.001
	4th and 5th (least deprived)	1813 (4.4)	127 (2.4)	17.4 (14.6–20.6)	1		1	
	Missing	31 (0.1)	3 (0.1)					

*IMD* Index of Multiple Deprivation, *IQR* interquartile range

^a^Adjusted by the remaining socio-demographic variables (gender, ethnicity, IMD) and time variables (age, year)

**Fig. 4:**
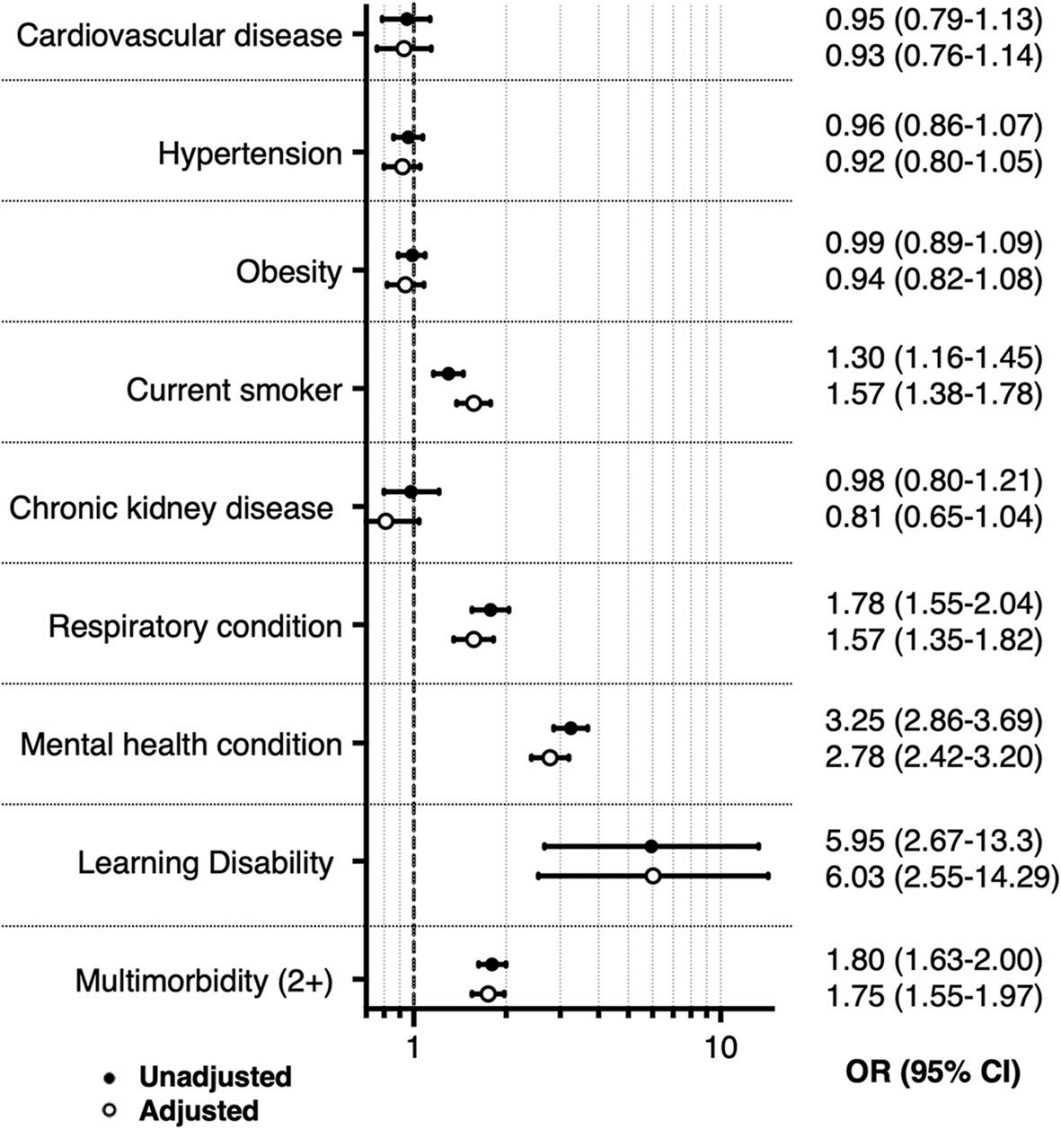
Distribution of clinical features and their association with referral into SP amongst people eligible for NDPP, cross-sectional study. Legend: See Additional File 1: Table S6 for information on the variables included in each adjusted model

Sixty-seven per cent (27,928) of people at high risk of T2D met the eligibility criteria for NDPP and were, therefore, included in our cross-sectional study. Of these people, 11% (3015) had been referred to SP, 10% (2899) to NDPP and 2% (518) to both services. As shown in Table 3, patients referred into SP were significantly more likely to be female (OR 1.99 (95% CI 1.78–2.24)) and socio-economically deprived (OR 2.12 (95% CI 1.56–2.88)) than those referred into NDPP, but less likely to be South Asian (OR 0.48 (95% CI 0.41–0.56)), black (OR 0.46 (95% CI 0.37–0.56)) or Chinese (OR 0.14 (0.07–0.27)). Supporting the findings from previous cohort studies, people diagnosed with mental health conditions (OR 3.25 (95% CI 2.86–3.69)) or multimorbidity (OR 1.80 (95% CI 1.63–2.00)) were also more likely to be referred into SP. Adjustments for relevant variables did not alter the results substantially (Fig. 4 and Additional file 1: Table S6).

**Table 3:** Distribution of socio-demographic characteristics amongst patients eligible for NDPP and their association with referral into SP compared to NDPP, cross-sectional study

Variables		No referral 21,483 (%)	Only SP *N* 3028 (%)	Only NDPP *N* 2899 (%)	Both *N* 518 (%)	OR (95% CI)	*P* value	OR^a^ (95% CI)	*P* value
Gender	Male	10,006 (46.6)	1182 (39.0)	1587 (54.7)	213 (41.1)	1		1	
	Female	11,476 (53.4)	1846 (61.0)	1311 (45.2)	305 (58.9)	1.38 (1.28–1.50)	<0.001	1.99 (1.78–2.24)	<0.001
	Missing	1 (0.01)	0	1 (0.1)	0				
Age median (IQR)		49 (39, 60)	53 (43, 63)	52 (44, 61)	54 (46, 64)	1.00 (0.99–1.01)	0.129	0.99 (0.99–1.01)	0.590
Ethnicity	White	5689 (26.5)	831 (27.4)	490 (16.9)	92 (17.8)	1		1	
	South Asian	9873 (46.0)	1465 (48.4)	1542 (53.2)	266 (51.4)	0.56 (0.49–0.64)	<0.001	0.48 (0.41–0.56)	<0.001
	Chinese	342 (1.6)	12 (0.4)	46 (1.6)	6 (1.2)	0.15 (0.08–0.29)	<0.001	0.14 (0.07–0.27)	<0.001
	Black	1664 (7.8)	259 (8.6)	289 (10.0)	58 (11.2)	0.53 (0.43–0.65)	<0.001	0.46 (0.37–0.56)	<0.001
	Arab	76 (0.4)	13 (0.4)	7 (0.2)	2 (0.4)	1.10 (0.43–2.76)	0.848	1.03 (0.41–2.62)	0.944
	Mixed/others	775 (3.6)	104 (3.4)	81 (2.8)	18 (3.5)	0.76 (0.55–1.03)	0.080	0.68 (0.50–0.93)	0.017
	Missing	3064 (14.3)	344 (11.4)	444 (15.3)	76 (14.7)				
IMD quintiles	1st and 2nd (most deprived)	19,092 (88.9)	2838 (93.7)	2631 (90.7)	477 (92.1)	1.78 (1.33–2.39)	<0.001	2.12 (1.56–2.88)	<0.001
	4th and 5th (least deprived)	1115 (5.2)	74 (2.4)	122 (4.2)	17 (3.3)	1		1	
	Missing	19 (0.1)	3 (0.1)	4 (0.1)	0 (0.0)				

OR comparing referral into only SP with referral into only NDPP (baseline category)

*IMD* Index of Multiple Deprivation, *IQR* interquartile range, *NDPP* NHS Diabetes Prevention Programme

^a^Adjusted by the remaining socio-demographic variables (gender, age, ethnicity, IMD)

### How did social prescribing contribute to meeting the complex health and social needs of people at high risk of type 2 diabetes? Qualitative findings

We identified the following four mechanisms through which SP operated to reach high-risk patients with greatest health and social need.

#### Accessible social prescribing: type 2 diabetes prevention as an inclusive and proactive care process

Unlike NDPP, SP had broad eligibility criteria (any patient 18+ and registered with a local GP could be invited). Lack of requirements for tests or medical assessments prior to a referral made the service easier to consider by referrers during routine consultations: “there’s no restrictions. We don’t have like; your blood pressure has to be this. Your weight has to be this. We can just refer” [Nurse 01.8]. Although patients were not necessarily referred based on their T2D risk, they often ended up accessing services relevant to its prevention: “[…] he was pre-diabetic and he was very, very overweight, which wouldn’t surprise me because often these conditions go together. Through [name of physical activity programme] and the support he got, he lost a lot of weight. His sugar levels were much more normal, things like that. I think that’s a good example of where he wasn’t actually there for the diabetes side but actually it really helped him more generally” [VCS 03.9].

Our study also revealed that patients often lacked confidence and felt guilty or helpless to reach out and access the services they required. As expressed by a patient referred into SP: “I know how to contact her [the link worker] but you know, […] I don’t want to feel that you know – I feel a bit guilty really that I didn’t follow up on a lot of the information that she gave me” [SPU 04.1]. Additional support was often needed to help them navigate (and reach) SP. Strategies included scheduling regular follow-ups with link workers, filling in referral forms in primary care (instead of signposting or encouraging self-referrals) or creating friendly and welcoming environments in the VCS with the help of volunteers, buddy systems and/or information packages, amongst others. However, the relationship between the provision of support and patients’ access or engagement was non-linear and hence unpredictable. Patients often failed to respond, did not turn up to sessions or refused to carry on despite these supportive environments. Making services accessible in such situations often relied on providers’ capacity (and willingness) to be tenacious and attentive: “She persevered, even though there would have been times when I did not pick up that phone and I did not want to talk to [name of link worker] or anyone. Yet again she would try and she would always inform me that ‘I could not get hold of you today, I will try in another five or six days’ time’ and she always kept her promise” [SPU 04.3]. Accessibility was no longer a static service attribute, but rather a proactive (*creative* and *ongoing*) process through which providers tried to overcome existing barriers and find ways to bring services closer to the patient.

#### Holistic social prescribing: type 2 diabetes prevention as a dynamic and personalised practice

Providers throughout the SP referral pathway proactively explored patients’ wider socio-economic circumstances in search of concerns influencing their wellbeing and clinical presentations. Open conversations led to diverse courses of action, depending on the identified priorities. This broadened the scope and understanding of T2D prevention beyond lifestyle recommendations to also include services related to employment, housing or welfare advice, amongst others: “It’s not just about ‘I want to change my diet’, it will be looking at the barriers to them changing the diet. We might refer for the diet and exercise classes, but we’ll also refer to English classes and things like that” [Nurse 01.8]. Providers tried to widen and diversify their service remit to better accommodate patients’ multiple, intertwined needs (e.g. by providing in-house legal advice alongside physical activity programmes, up-skilling link workers on relevant domains (such as health coaching, welfare advice) or even bringing welfare advisors and lifestyle programmes into GP practices): “for example on a Thursday it’d be the advisor here and the receptionist would say, come and see the advisor in the surgery at that time. They’d make an appointment with them in the same way you’d make an appointment with the nurse. That’s excellent service” [VCS 03.9].

Instead of adhering to established, pre-defined ways of working, we identified joint attempts to work around patients’ complex life circumstances and adapt services accordingly. This involved prioritising patients’ context and the provision of support over the specific content and consistency of lifestyle recommendations: “It’s not specifically saying, this is what you must do for cholesterol. This is what you must do for diabetes. Very often the advice is the same anyway. […] the issue is not about the actual specific condition, it’s more about getting that peer support to help that person manage whatever’s going on for them in their life” [VCS 03.9]. Some patients needed “taking” (“I took patients on walking groups [...], just so that they go. […] I’ve taken a patient to an ESOL class because she didn’t want to go alone. So, I just took her to the first one” [LW 02.5]), while others needed to be listened to (“Sometimes also we receive referrals where they do not want any help; all they want is someone to listen to them” [LW 02.8]) or somebody to whom they could feel accountable (“I just needed somebody in a way to whom I could be answerable, if that sounds strange” [SPU 04.4]). Some patients preferred medicalised preventative approaches (where information about disease risk and anthropometric measurements were considered), while others responded better to “subtle” (opportunistic) lifestyle recommendations or regular follow-ups by health care assistants in primary care (instead of being referred into community-based lifestyle programmes).

#### Sustained social prescribing: type 2 diabetes prevention as an ongoing and unpredictable struggle

Patients reported that their capacity to follow a “healthy” lifestyle fluctuated over time, highly conditioned by the amount and consistency of support they received. Many patients had tried different weight management or physical activity programmes, managed to lose weight while being supported and then relapsed as the intensity of interventions decreased. Critically, the lack of a long-lasting response was interpreted by patients as a personal failure rather than a deficiency of the services in place or a consequence of underlying structural constraints (e.g. poverty, food insecurity, obesogenic environments, etc.): “when I did the [name of weight management programme] I’d gone down to 97kgs and that’s the best I ever did in my entire life. In the entire 35 years of my life that was the best I’d done and I felt great and I was a good size 18 and I was so happy. Then within stopping that programme because it’s again, for me, I think I lack and I don’t think it’s the services, for me I start something but […] I never sustain that, I never maintain anything. So, […] within a year I put all that back on plus more. So, it’s been very difficult” [SPU 04.3].

Regular services, conversely, allowed for the provision of ongoing support. Patients could build on previous work and share the burden of (and, hence, better cope with) critical social and health constraints: “I just felt a little bit better that I’m not dealing with this on my own” [SPU 04.3]. Continuity of care allowed providers to monitor patients’ progress (or lack thereof) and adapt their approach and next steps accordingly. “Testing and trying” involved making the erratic nature of prevention, as well as the limitations of existing interventions explicit beforehand. Knowing that interventions could fail, be insufficient or inappropriate prevented unrealistic expectations and shifted the responsibility of any potential failure from the individual to the intervention: “So, I think what the prescriber was saying was ‘my resources are limited and you could go to this walk-in therapy and hate it and that’s okay, you’d come back and tell me’. But I think so often people are referred to a social prescriber and they take up one offer and that doesn’t work and so they think nothing will work” [SPU 04.4].

Ongoing services allowed for the development of meaningful (“therapeutic”, “trustful”) relationships with patients, across sectors and amongst attendees in group sessions: “a lot of people have been coming for 10 plus years, so they’ve built up these really good bonds with each other. So, not only are we looking out for them but they’re looking out for each other” [R1 VCS 03.2]. Patients were made aware that “there [was] somebody there for them” [LW 02.5], which proved reassuring and had therapeutic effects by itself: “just knowing that there’s people available for us” [SPU 04.2]. It counteracted feelings of helplessness and mistrust towards a system that had previously let them down (“if [the service] stops it’s like, ‘oh they’re the same as everyone’.” [VCS 03.8]) and provided some stability within a context of great service and staff turnover (both within the health sector and the VCS): “with many services in the community coming and going, […] even in the NHS as well, programmes with different names each year, I think there’s real benefit in having someone stable, by having a known person in a GP surgery that they can come to whenever there is a change in their life situation because that often provides that window of opportunity to really change something” [LW 02.2].

#### Integrated social prescribing: type 2 diabetes prevention as a locally embedded and joint endeavour

Bidirectional communication across sectors and among professionals allowed providers to develop greater knowledge on patients’ needs, “build on other’s work” and deliver consistent care. Clinicians became increasingly aware of patients’ “underlying story” and able to adapt care practices accordingly because of the information that link workers had shared: “I quickly realised how much the GPs are really struggling to know what the story is behind the medical record and […] just by sharing a few lines, […] ‘well actually this person had an accident last year or lost their job’, just very simple things that make us able to humanise people and their choices” [LW 02.2]. Patients’ medical notes provided link workers with “a two-sided approach” and relevant “context” (“background information”) over which they could build their assessment and recommendations. Similarly, VCS organisations often received key information with or prior to a referral on how best to support a patient by avoiding specific “triggers” that had already been disclosed. Communication channels across and within sectors were also used to flag those patients requiring closer, urgent attention (“Usually, they give us one- or two-lines feedback […] There’s also something on the records […] and, for very specific people that I’m maybe a bit more worried about, I may just ask them to let me know what has happened” [GP 01.1]) and/or reach out if they had not been contacted. Service providers became effective advocates by ensuring patients were not left behind and received the support they required: “When I felt depressed about my weight and not getting any contact from the weight management programme, [name of link worker] was right on it sending emails and making telephone calls, whilst I was on the phone by the way, and getting in touch with them and saying ‘look, I’ve got a patient here named… she’s waited for that number of weeks, why has nothing come through?’ So, I felt like the support was first-hand” [SPU 04.1].

Integration (and embeddedness) within the local community and primary care system also enhanced the scope and responsiveness of services. Patients at risk of T2D ended up accessing physical activity programmes that would have been difficult to locate had it not been for the SP network (“any kind of exercise programmes that we have running or any health sessions we try and send out to the GPs and that’s where a lot of our referrals came from” [VCS 03.2]). Link workers informed the design of VCS activities (e.g. lifestyle programmes, physical activity sessions) by sharing relevant information on identified needs and facilitated their development in local GP practices: “we would often run services at GP surgeries to increase accessibility. For example, [name of GP practice] has a room, we’d meet in there” [VCS 03.9]. Clinicians and link workers often worked together (and learnt from each other) to support patients with complex needs: “if you’re having any difficult patients, challenging, you can bounce off or you can say, ‘How are you dealing with that patient?’ Maybe in a different way and you see whether you can make sense of it or not” [LW 02.4]. This not only led to greater service appropriateness but also strengthened the local community by creating new partnerships and opportunities: “there’s an opportunity here for us to develop physical and social activities together. So it’s about doing things together and one of the – we use local residents who’ve become qualified instructors, we try to keep everything local” [VCS 03.8].

### Synthesis: primary care-based social prescribing as a means for successful individual-level type 2 diabetes prevention

**Fig. 5:**
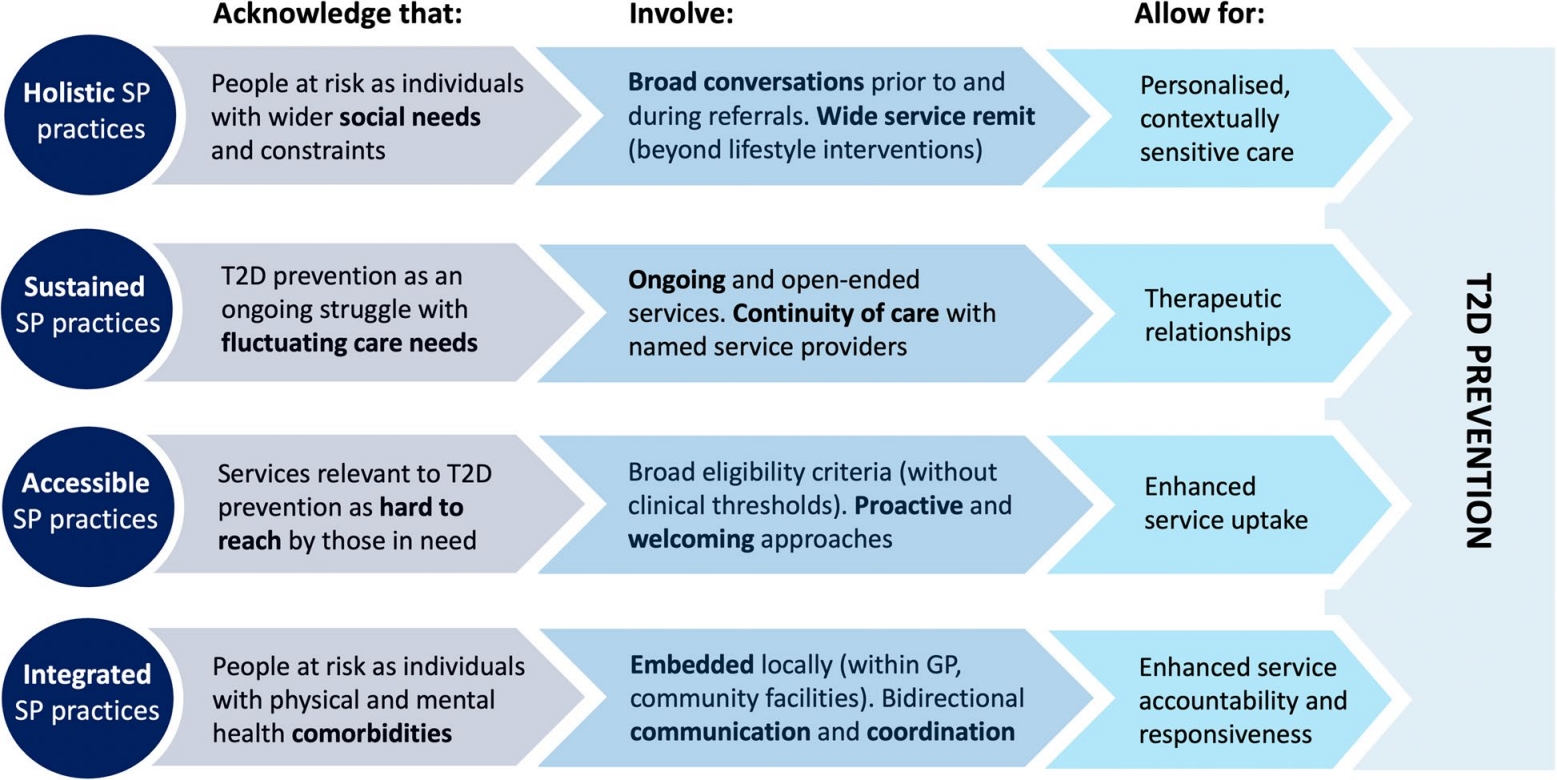
Synthesis of study findings. How SP operates to deliver T2D prevention

Figure 5 brings together quantitative and qualitative findings by illustrating why and how primary care-based SP contributed to individual-level T2D prevention approaches relevant to patients in greatest social and health need. The four dimensions (represented as four horizontal lines in Fig. 5) summarise our final CMOCs, which are described in more detail in Additional file 3: Fig. S1.

Highly supportive (*proactive*, *welcoming*) environments and broad (*inclusive*) referral criteria made SP easier to access than NDPP by high T2D risk individuals in greatest health and social need (CMOC1). *Holistic* practices involved gaining understanding of these underlying health and social constraints (including what they meant for people at high risk) and providing tailored care and services accordingly. This was often achieved by holding broad conversations with patients and widening the scope and remit of available services (CMOC2). Following a “healthy” lifestyle proved highly demanding for patients at high risk (especially insofar as underlying drivers persisted) and hence benefitted from *sustained* (ongoing and open-ended) support by known and trustful service providers (CMOC3). Our quantitative data also clearly showed that people at risk of T2D with multiple physical and mental health morbidities were more likely to be referred to SP than NDPP and our qualitative data suggests that this likely reflects the integrated delivery of SP including cross-referrals, connected and seamless care within primary care and across sectors (CMOC4).

The identified *mechanisms* were interconnected and mutually dependent, meaning that they only became possible (and led to significant outcomes) as the rest of the dimensions co-existed. *Holistic* practices, for instance, were more than a set of questions asked in one consultation prior to a referral. They came about only over time (through *sustained* practice) as providers got acquainted with (and gained understanding of) the local population, their community and the specific patient involved. Similarly, providers were able to deliver proactive care and make sure services were *accessed* only through regular follow-ups and timely feedback from other practitioners (“I know she started because the [name of VCS organisation] updated me, so she has started” [LW 02.11]). *Integrated* SP facilitated a greater understanding of patients’ circumstances, enhanced service responsiveness and allowed to address their needs *holistically*. Yet, far from representing a static structural dimension, it relied on trustful interpersonal relations across providers and hence required time (*sustained* encounters) to be developed: “I’ve built such a good relationship with [VCS organisations], and it’s because you’ve been doing it for so long, you get to know people on the team as well because you’re backwards and forwards with emails” [LW 02.5].

## Discussion

This mixed-method study, in a multi-ethnic, inner-city locality with high levels of deprivation, systematically illustrated how accessible, holistic, sustained and integrated SP practices in primary care appeared to support the delivery of individual-level T2D preventative approaches relevant (and available) to those in greatest health and social need.

The qualitative study showed that lack of disease (or diabetes)-specific eligibility criteria simplified the referral of patients to SP during routine, busy primary care consultations, which resulted in increased access of high T2D risk individuals to health promotion and wellbeing activities (all relevant to T2D prevention). This inclusive approach to T2D prevention seems particularly relevant given the low accuracy (and inconsistencies) of current pre-diabetes screening strategies (whereby patients may receive an incorrect diagnosis while others be falsely reassured and not offered any intervention [47]) and the generalised merit of healthier diet and exercise (which are likely to benefit most patients in multiple aspects of health, not just their risk of developing diabetes [48]). Our research suggests that *accessibility* in T2D prevention (and SP) can be best understood as a twin process of identifying patients’ needs and conditions (instead of risk levels) while finding ways to make services readily available to them, which often entailed ensuring highly supportive environments.

We showed how patients’ health-related behaviours (and consequent T2D risk) are contingent and socially patterned [49, 50], and therefore amenable to change insofar as interventions are within patients’ material reach, familiar to their existing social world and relevant to their life circumstances [51]. The literature has shown mixed responses to similar T2D preventative strategies. For example, while some studies found that regular feedback regarding risk level prompted attendance to lifestyle interventions and successful behavioural change [52], others reported the opposite reaction, whereby obtaining new knowledge specific to patients’ own risk elicited negative feeling and prevented further attendance [53, 54]. These inconsistencies confirm that different strategies work for different people and suggest (in line with our study findings) that a personalised (*holistic*) approach which takes into consideration patients’ specific (and changing) characteristics, priorities, expectations and circumstances might be better suited to deliver effective individual-level T2D prevention [22, 55].

The benefit of *sustained* approaches in T2D prevention has been widely acknowledged in the literature [22, 55]. Participating in interventions often acts as a relevant motivator for change and may provide “relief” (and sense of fulfilment) for being committed (and striving) towards betterment, regardless of the outcome [56]. Following healthy lifestyle recommendations may also be lived as an ongoing “struggle” (given underlying, persistent constraints) and hence benefit from continuous support [56]. Trustful relationships with service providers (enabled through and within ongoing interventions) have also been found to help patients make informed decisions about their health and contribute to healthy lifestyle maintenance. Critically, short-term interventions may lead to feelings of “frustration”, “failure” or “guilt” amongst participants for not achieving the intended (though arguably unrealistic) outcomes [48, 51].

Our study, in line with published literature, revealed that high-risk patients often suffered from co-morbidities and multimorbidity, which made them require (and access regularly) primary health care. *Integrating* SP and T2D prevention into routine primary care allowed for opportunistic health promotion advice to those who might have contacted the GP regarding a different (yet, coexistent) concern [55, 57]. It also encouraged to think of and practice the promotion of a healthy lifestyle as an ongoing and incremental process, instead of a one-off intervention [58]. Lastly, relying on community-based, local organisations (as opposed to external private providers, as was the case in NDPP) contributed to strengthening the local community [53, 59]. Services became “more than a place” in which patients were seen (or referred into) to be also conceived as “community spaces” where meaningful social connection and community action could engender (whose benefits may transcend T2D prevention) [60].

Our study suggests that SP may offer an opportunity for individual-level T2D prevention to become more inclusive, personalised and long-term. Interestingly, this was achieved through the very same interlinked mechanisms that define the strength and essence of primary care (namely, comprehensiveness, continuity of care, contact accessibility and coordination, as defined by Starfield [61]). This parallelism reminds us that SP relevant to T2D prevention both requires and results in a strengthened primary care system. Such practices, however, did not happen in a cultural or historical vacuum. They were enacted within (and shaped by) an environment characterised by a long (and proud) history of partnership between the health sector and local voluntary, community and faith groups. In adapting and accommodating practices together (over time), SP became recognisable and relevant to all concerned.

## Strength and limitations

To our knowledge, this is the first study exploring the potential of SP in the prevention of T2D. We identified key ingredients that contributed to explaining how (and why) SP succeeded in reaching people at high risk of T2D with greatest health and social vulnerability, while overcoming some of the limitations described in existing T2D prevention programmes. Another key strength of this study is the theoretically grounded, methodologically pluralistic design [62], derived from previous realist [21] and discourse analysis [9] reviews of primary care-based SP literature. The use of a realist approach and the combination of qualitative and quantitative methodologies allowed us to define rich, concrete, context-dependent exemplars of “good” practice in SP and T2D prevention and develop useful recommendations for policy and practice.

The study has limitations. It was confined to a single locality with a particular history and ethos of social engagement and provider innovation. Findings might not, therefore, be applicable elsewhere—in particular the close and productive working relationships between public and third-sector organisations in our study site cannot be expected to occur everywhere. Quantitative data were restricted to referrals into SP (and/or NDPP) and did not, therefore, capture the extent to which patients actually engaged with SP or NDPP, the type and duration of activities accessed, subsequent actions or clinical outcomes. Qualitative findings, however, helped to mitigate these constraints by providing rich context and in-depth explanations, including the key ingredients for potentially successful individual-level T2D prevention and detailed accounts of how (and why) programmes may (or may not) work. Our quantitative dataset also showed 24% of missing ethnicity data, which although may have altered the real ethnic distribution of our cohort, is in line with the proportion of missingness described in published electronic health records studies [63]. Lastly, pandemic restrictions involved adaptations of both the intervention(s) (e.g. remote SP and NDPP sessions) and data collection strategies (e.g. holding some of the ethnographic observations and qualitative interviews remotely). This might have biased our sample towards less deprived and digitally literate participants. We sought to mitigate this by offering phone as well as video interviews, adapting interview schedules to meet individual circumstances and undertaking face-to-face interviews as soon as it was permissible and safe to do so.

## Implications for practice and policy

This realist evaluation generates a framework (Fig. 5) that could contribute to guiding the development, implementation and evaluation of SP programmes relevant to the prevention of T2D in communities at high risk. Building on rich, empirical data we defined “best practice(s)” in SP relevant to T2D prevention and identified the conditions (“key ingredients”) that need to be in place to facilitate this (and why).

Our study revealed the potential of accessible, holistic, sustained and integrated SP practices in T2D prevention. Critically, such approaches are not acknowledged by (and even seem to contradict) overarching SP health policy discourses and arrangements, which emphasise and prioritise the commissioning of short-term, motivational interventions [9]. Our study supports a revision of exiting SP health policy and commissioning strategies, so that they facilitate (and encourage) personalised, inclusive and long-term preventative approaches in primary care and the VCS. Study findings may also provide valuable insights into how to enhance the reach and equity of access of existing NHS T2D preventative strategies, such as NDPP (especially where a social gradient exists in those accessing and gaining benefit from the service) or develop adapted interventions that combine or integrate elements from SP and NDPP.

## Conclusions

Our research revealed the need (and merit) of an alternative framing of individual-level T2D prevention: as personalised, long-term and inclusive *practices* rather than standardised, short-term and targeted *interventions* (such as NDPP). SP proved ideally placed to deliver this where practitioners, providers and commissioners worked collectively to achieve holistic, sustained, accessible and integrated services. The wider contexts in which these practices developed proved, however, far from neutral. Existing organisational arrangements, priorities and routines shaped providers’ capacity (and willingness) to deliver SP relevant to patients at high risk. Additional research is being undertaken by the researchers involved in this study to investigate existing barriers and enablers for the delivery of accessible, holistic, sustained and/or integrated SP practices in primary care. Further research will also be critical to ascertain whether (and if so, to what extent) such practices contribute to reducing patients’ overall risk of developing T2D and to support appropriate delivery and roll-out in different settings.

## Supplementary Information

**Additional file 1. **Quantitative research design and results. **Table S1.** Overview of quantitative research design. **Table S2.** Distribution of clinical characteristics within the total study population and their association with referral into SP. **Table S3.** Distribution of clinical characteristics amongst people at high risk of T2D and their association with referral into SP. **Table S4.** Study year, age, and their association with referral to SP within the study population. **Table S5.** Study year, age, and their association with referral to SP amongst patients at high risk of T2D. **Table S6.** Association of clinical characteristics with referral into only SP compared to referral into only NDPP amongst patients eligible for NDPP.

**Additional file 2. **Qualitative data sources and sample characteristics. **Table S1.** Qualitative data sources and their contribution to the study. **Table S2.** Characteristics of SP users interviewed. **Table S3.** Characteristics of link workers interviewed. **Table S4.** Characteristics of primary care clinicians interviewed. **Table S5.** Characteristics of VCS members interviewed.

**Additional file 3. **COMCs developed in the realist mixed-methods evaluation. **Figure S1.** Realist evaluation COMCs.

## Data Availability

The datasets used and/or analysed during the current study are available from the corresponding author on reasonable request.

## Abbreviations

NDPP: NHS Diabetes Prevention Programme
SP: Social prescribing
T2D: Type 2 diabetes
VCS: Voluntary and community sector
