# Supplementary material for: Unravelling the potential of social prescribing in individual-level type 2 diabetes prevention: a mixed-methods realist evaluation
Source: BMC Med. 2023 Mar 13;21:91. doi: 10.1186/s12916-023-02796-9 (PMC10008720; doi:10.1186/s12916-023-02796-9)
Supplement: Supplementary file 1 — Additional file 1. Quantitative research design and results. Table S1. Overview of quantitative research design. Table S2. Distribution of clinical characteristics within the total study population and their association with referral into SP. Table S3. Distribution of clinical characteristics amongst people at high risk of T2D and their association with referral into SP. Table S4. Study year, age, and their association with referral to SP within the study population. Table S5. Study year, age, and their association with referral to SP amongst patients at high risk of T2D. Table S6. Association of clinical characteristics with referral into only SP compared to referral into only NDPP amongst patients eligible for NDPP. [file 12916_2023_2796_MOESM1_ESM.docx]

**Additional File 1. Quantitative research design and results**

**Table S1.** Overview of quantitative research design

| **Study design** | **Study population** | | | **Main variables** | |
| --- | --- | --- | --- | --- | --- |
|  | **Entry date** | **Exit date** | **Excluded** | **Outcome** | **Main exposure** |
| Cohort study (1) | People eligible for SP (18+ and active GP registration in Tower Hamlets CCG). Latest date of   - Registration with practice - Beginning of study (1/12/2016) | Earliest date of   - SP referral (outcome) - Death or deregistration - End of study (14/2/2022) | Patients with known T1D or T2D | SP referral | T2D high risk, defined by:   - GDM, - QDScore >= 20, - FBG 5.5-6.9 mmol/L, - HBA1c 42-47 mmol/mol, - Diagnosis of NDHG, - Referral into NDPP |
| Cohort study (2) | People at high risk of T2D:   - Earliest date of the diagnosis of T2D high risk status - Latest date of practice registration or beginning of study (1/12/2016) if diagnosed with high risk T2D before the study started | Earliest date of   - SP referral (outcome) - Death or deregistration - End of study (14/2/2022) | Patients with known T1D or T2D | SP referral | - Socio-demographics (age, gender, ethnicity, SES) - Clinical (CVD, HYT, obesity, smoking, CKD, respiratory condition, mental health problem, learning disability, multimorbidity) |
| Cross sectional study | People at high risk of T2D eligible for NDPP:   - Age between 18 and 79, and: - GDM, - FPG 5.5-6.9 mmol/L, - HBA1c 42-47 mmol/mol, or - Diagnosis of non-diabetic hyperglycaemia | | Patients with known T1D or T2D | - SP only - NDPP only - None - Both | - Socio-demographic and clinical, as described above |

SP: Social Prescribing. NDPP: NHS Diabetes Prevention Programme. CCG: Clinical Commissioning Group. GDM: Gestational Diabetes Mellitus. FBG: Fasting Blood Glucose. CVD: Cardiovascular disease. HYT: hypertension. CKD: Chronic Kidney Disease. T1D: type 1 diabetes. T2D: type 2 diabetes.

**Table S2**. Distribution of clinical characteristics within the total study population and their association with referral into SP

| Variables | Total  N 447,360 (%) | Events (SP)  N 15,454 (%) | Rates per 1000 P/Y | Crude RR  (95% CI) | P value | Adjusted RR (95% CI) | P value |
| --- | --- | --- | --- | --- | --- | --- | --- |
| Cardiovascular*^a^ (IHD, PAD or STIA) | 4,926 (1.1) | 817 (5.3) | 43.1 (40.2-46.1) | 4.67 (4.35-5.01) | <0.001 | 1.42 (1.30-1.54) | <0.001 |
| Hypertension*^b^ | 19,684 (4.4) | 2,540 (16.4) | 32.1 (30.9-33.4) | 3.79 (3.64-3.96) | <0.001 | 1.20 (1.14-1.27) | <0.001 |
| Obesity* | 68,862 (15.4) | 6,008 (38.9) | 22.3 (21.8-22.9) | 3.15 (3.05-3.26) | <0.001 | 1.83 (1.76-1.91) | <0.001 |
| Current smoker* | 106,379 (23.8) | 5,548 (35.9) | 13.7 (13.3-14.0) | 1.65 (1.60-1.71) | <0.001 | 1.69 (1.63-1.75) | <0.001 |
| Chronic Kidney Disease*^c^ | 5,553 (1.2) | 775 (5.0) | 34.5 (32.2-37.0) | 3.72 (3.46-3.99) | <0.001 | 0.98 (0.90-1.07) | 0.723 |
| Respiratory*^d^ (asthma or COPD) | 40,996 (9.2) | 2,954 (19.1) | 18.6 (18.0-19.3) | 2.16 (2.07-2.25) | <0.001 | 1.68 (1.60-1.75) | <0.001 |
| Mental health* (mild to severe) | 45,241 (10.1) | 5,389 (34.9) | 32.8 (31.9-33.7) | 4.70 (4.54-4.85) | <0.001 | 3.44 (3.31-3.57) | <0.001 |
| Learning Disability* | 1,184 (0.3) | 268 (1.7) | 56.4 (50.0-63.6) | 5.94 (5.27-6.70) | <0.001 | 5.33 (4.69-6.06) | <0.001 |
| Multimorbidity* (+2) | 38,024 (8.5) | 5,559 (36.0) | 37.5 (36.6-38.5) | 5.53 (5.35-5.71) | <0.001 | 2.90 (2.79-3.02) | <0.001 |
| **High risk of T2D***^c^ | 41,378 (9.3) | 5,226 (33.8) | 30.8 (29.9-31.6) | 4.31 (4.17-4.46) | <0.001 | 1.33 (1.27-1.39) | <0.001 |

*Adjusted by variables in level 1: age-band, gender, ethnicity, IMD/SES and year; *^a^Adjusted by variables in level 1 plus obesity, hypertension, high risk of T2D, current smoker and CKD; *^b^Adjusted by variables in level 1 plus obesity and current smoker; *^c^Adjusted by variables in level 1 plus obesity, hypertension, current smoker; *^d^Adjusted by variables in level 1 plus current smoker. SP: Social Prescribing. IHD: Ischemic Heart Disease. PAD: Peripheral Arterial Disease. STIA: Stroke and Transient Ischemic Attack. COPD: Chronic Obstructive Pulmonary Disease.

**Table S3**. Distribution of clinical characteristics amongst people at high risk of T2D and their association with referral into SP

| Variables | Total  N 41,378 (%) | Events (SP)  N 5,226 (%) | Rates per 1000 P/Y | RR (95% CI) | P value | Adjusted RR* (95% CI) | P value |
| --- | --- | --- | --- | --- | --- | --- | --- |
| Cardiovascular*^a^ (IHD, PAD or STIA) | 3,353 (8.1) | 586 (11.2) | 43.8 (40.4-47.4) | 1.43 (1.31-1.55) | <0.001 | 1.32 (1.19-1.45) | <0.001 |
| Hypertension*^b^ | 12,483 (30.2) | 1,887 (36.1) | 36.3 (34.7-37.9) | 1.22 (1.16-1.29) | <0.001 | 1.07 (0.99-1.14) | 0.054 |
| Obesity* | 23,433 (56.6) | 3,276 (62.7) | 35.2 (34.1-36.5) | 1.30 (1.23-1.37) | <0.001 | 1.29 (1.20-1.38) | <0.001 |
| Current smoker* | 13,029 (31.5) | 1,878 (35.9) | 36.1 (34.5-37.8) | 1.22 (1.15-1.29) | <0.001 | 1.40 (1.31-1.49) | <0.001 |
| Chronic Kidney Disease*^c^ | 3,559 (8.6) | 517 (9.9) | 34.9 (32.0-38.0) | 1.11 (1.01-1.22) | 0.024 | 0.90 (0.81-1.01) | 0.053 |
| Respiratory*^d^ (asthma or COPD) | 6,428 (15.5) | 1,177 (22.5) | 46.6 (44.0-49.3) | 1.60 (1.50-1.71) | <0.001 | 1.46 (1.36-1.57) | <0.001 |
| Mental health* (mild to severe) | 7,846 (18.9) | 1,794 (34.3) | 59.1 (56.4-61.9) | 2.31 (2.18-2.45) | <0.001 | 2.21 (2.07-2.35) | <0.001 |
| Learning Disability* | 256 (0.6) | 69 (1.3) | 75.8 (59.9-95.9) | 2.41 (1.90-3.05) | <0.001 | 2.79 (2.18-3.58) | <0.001 |
| Multimorbidity* (+2) | 16,856 (40.7) | 3,034 (58.1) | 44.8 (43.3-46.5) | 1.98 (1.88-2.10) | <0.001 | 1.87 (1.75-1.99) | <0.001 |

*Adjusted by variables in level 1: age-band, gender, ethnicity, IMD/SES and year; *^a^Adjusted by variables in level 1 plus obesity, hypertension, current smoker and CKD; *^b^Adjusted by variables in level 1 plus obesity and current smoker; *^c^Adjusted by variables in level 1 plus obesity, hypertension, current smoker; *^d^Adjusted by variables in level 1 plus current smoker. SP: Social Prescribing. IHD: Ischemic Heart Disease. PAD: Peripheral Arterial Disease. STIA: Stroke and Transient Ischemic Attack. COPD: Chronic Obstructive Pulmonary Disease.

**Table S4**. Study year, age, and their association with referral to SP within the study population

|  | | Events SP | P/Y | Rates/1000 P/Y  (95% CI) | Crude RR (95% CI) | P value |
| --- | --- | --- | --- | --- | --- | --- |
| Age-band | 18-29 | 3517 | 614.8 | 5.7 (5.5-5.9) | 1 | |
|  | 30-39 | 3919 | 555.1 | 7.1 (6.8-7.3) | 1.23 (1.18-1.29) | <0.001 |
|  | 40-49 | 3077 | 221.3 | 13.9 (13.4-14.4) | 2.43 (2.32-2.55) | <0.001 |
|  | 50-59 | 2242 | 105.6 | 21.2 (20.4-22.1) | 3.71 (3.52-3.91) | <0.001 |
|  | 60-69 | 1401 | 53.1 | 26.4 (25.0-27.8) | 4.61 (4.33-4.91) | <0.001 |
|  | =>70 | 1298 | 54.3 | 23.9 (22.6-25.2) | 4.18 (3.92-4.45) | <0.001 |
| Year | 1^st^ (2017) | 5057 | 412.5 | 12.3 (11.9-12.6) | 1.48 (1.33-1.65) | <0.001 |
|  | 2^nd^ (2018) | 2651 | 349.7 | 7.6 (7.3-7.9) | 0.91 (0.82-1.03) | 0.120 |
|  | 3^rd^ (2019) | 2484 | 310.5 | 8.0 (7.7-8.3) | 0.97 (0.86-1.08) | 0.537 |
|  | 4^th^ (2020) | 2627 | 266.6 | 9.9 (9.5-10.2) | 1.19 (1.06-1.33) | 0.003 |
|  | 5^th^ (2021) | 2295 | 224.0 | 10.2 (9.8-10.7) | 1.24 (1.10-1.39) | <0.001 |
|  | 6^th^ (2022 Q1) | 340 | 41.0 | 8.3 (7.5-9.2) | 1 | |

SP: Social Prescribing.

**Table S5.** Study year, age, and their association with referral to SP amongst patients at high risk of T2D

|  | | Events SP | P/Y | Rates/1000 P/Y  (95% CI) | Crude RR (95% CI) | P value |
| --- | --- | --- | --- | --- | --- | --- |
| Age-band | 18-29 | 199 | 7.8 | 25.5 (22.2-29.3) | 1 | |
|  | 30-39 | 699 | 26.0 | 26.9 (25.0-29.0) | 1.06 (0.90-1.24) | 0.506 |
|  | 40-49 | 1376 | 47.1 | 29.2 (27.7-30.8) | 1.15 (0.99-1.33) | 0.071 |
|  | 50-59 | 1201 | 36.4 | 33.0 (31.2-34.9) | 1.30 (1.12-1.51) | 0.001 |
|  | 60-69 | 869 | 24.1 | 36.1 (33.7-38.5) | 1.42 (1.21-1.65) | <0.001 |
|  | =>70 | 882 | 23.2 | 38.0 (35.6-40.6) | 1.49 (1.28-1.74) | <0.001 |
| Year | 1^st^ (2017) | 1426 | 39.1 | 36.6 (34.7-38.5) | 1.24 (1.04-1.48) | 0.016 |
|  | 2^nd^ (2018) | 909 | 35.5 | 25.6 (24.0-27.3) | 0.87 (0.73-1.04) | 0.129 |
|  | 3^rd^ (2019) | 981 | 32.3 | 30.4 (28.6-32.4) | 1.03 (0.86-1.24) | 0.724 |
|  | 4^th^ (2020) | 991 | 28.6 | 34.7 (32.6-36.9) | 1.18 (0.98-1.41) | 0.075 |
|  | 5^th^ (2021) | 782 | 24.6 | 31.8 (29.7-34.2) | 1.08 (0.90-1.30) | 0.399 |
|  | 6^th^ (2022 Q1) | 134 | 4.6 | 29.4 (24.8-34.9) | 1 | |

SP: Social Prescribing.

**Table S6.** Association of clinical characteristics with referral into only SP compared to referral into only NDPP amongst patients eligible for NDPP

| **Referral into SP** | **OR (95% CI)** | **P value** | **OR (95% CI)*** | **P value** |
| --- | --- | --- | --- | --- |
| Cardiovascular*^a^ (IHD, PAD, or STIA) | 0.95 (0.79-1.13) | 0.533 | 0.93 (0.76-1.14) | 0.479 |
| Hypertension*^b^ | 0.96 (0.86-1.07) | 0.473 | 0.92 (0.80-1.05) | 0.201 |
| Obesity***** | 0.99 (0.89-1.09) | 0.785 | 0.94 (0.82-1.08) | 0.377 |
| Current smoker***** | 1.30 (1.16-1.45) | <0.001 | 1.57 (1.38-1.78) | <0.001 |
| Chronic Kidney Disease*^c^ | 0.98 (0.80-1.21) | 0.866 | 0.81 (0.65-1.04) | 0.099 |
| Respiratory*^d^ (asthma or COPD) | 1.78 (1.55-2.04) | <0.001 | 1.57 (1.35-1.82) | <0.001 |
| Mental health* (mild to severe) | 3.25 (2.86-3.69) | <0.001 | 2.78 (2.42-3.20) | <0.001 |
| Learning Disability* | 5.95 (2.67-13.3) | <0.001 | 6.03 (2.55-14.29) | <0.001 |
| Multimorbidity* (+2) | 1.80 (1.63-2.00) | <0.001 | 1.75 (1.55-1.97) | <0.001 |

OR comparing referral into only SP with referral into only NDPP (baseline category). **Adjusted by each other *Adjusted by variables in level 1: age-band, gender, ethnicity, IMD/SES and year; *^a^Adjusted by variables in level 1 plus obesity, hypertension, current smoker and CKD; *^b^Adjusted by variables in level 1 plus obesity and current smoker; *^c^Adjusted by variables in level 1 plus obesity, hypertension, current smoker; *^d^Adjusted by variables in level 1 plus current smoker. SP: Social Prescribing. NDPP: NHS Diabetes Prevention Programme. IHD: Ischemic Heart Disease. PAD: Peripheral Arterial Disease. STIA: Stroke and Transient Ischemic Attack. COPD: Chronic Obstructive Pulmonary Disease.
