# Supplementary material for: Unravelling the potential of social prescribing in individual-level type 2 diabetes prevention: a mixed-methods realist evaluation
Source: BMC Med. 2023 Mar 13;21:91. doi: 10.1186/s12916-023-02796-9 (PMC10008720; doi:10.1186/s12916-023-02796-9)
Supplement: Supplementary file 2 — Additional file 2. Qualitative data sources and sample characteristics. Table S1. Qualitative data sources and their contribution to the study. Table S2. Characteristics of SP users interviewed. Table S3. Characteristics of link workers interviewed. Table S4. Characteristics of primary care clinicians interviewed. Table S5. Characteristics of VCS members interviewed. [file 12916_2023_2796_MOESM2_ESM.docx]

**Additional File 2. Qualitative data sources and sample characteristics**

**Table S1.** Qualitative data sources and their contribution to the study

| **Data source(s)** | | **Sampling** | **Purpose** |
| --- | --- | --- | --- |
| Semi-structured interviews | 8 SP users at risk of T2D | Purposive: age, gender, ethnicity, diabetes risk status, degree of participation in SP | To investigate personal experiences, attitudes and perceptions in relation to SP and T2D prevention |
|  | 11 primary care clinicians | Purposive: professional profile, workplace, type of contract, seniority, sociodemographic characteristics (age, gender, ethnicity) |  |
|  | 11 link workers | Purposive: workplace, type of contract, time worked as link worker in local area, demographic characteristic (age, gender, ethnicity) |  |
|  | 13 VCS organisations | Purposive: source of funding, type of activities, community embeddedness |  |
| Observations | VCS physical activity programme  (12 hours) | Purposive: uptake and potential relevance for T2D prevention | To investigate how practices and activities unfolded in ‘naturally occurring’ community contexts |
|  | VCS weight management programme  (8 hours) |  |  |
|  | VCS meetings (10 hours) | Monthly VCS meetings held by local VCS organisations, LWs and community navigators |  |
|  | NDPP sessions (6 hours) | Privately delivered NHS T2D prevention programme |  |
| Documents | Related to the SP scheme, NDPP programme and VCS organisations (descriptions of activities on offer, target population, eligibility criteria, and evaluation reports). | | To provide historical and contextual insight about SP and NDPP |

SP: Social Prescribing. NDPP: NHS Diabetes Prevention Programme. T2D: type 2 diabetes. VCS: Voluntary and Community Sector. HCA: Health Care Assistants.

**Table S2.** Characteristics of SP users interviewed

| **Code** | **T2D status** | **Reason for referral** | **Participation** | **Link Worker** | **Age & Gender** | **Main language** | **Ethnicity** | **Mode** |
| --- | --- | --- | --- | --- | --- | --- | --- | --- |
| 04.1 | QDS> 20 | Weight management | Several LW visits and referrals | In-house | 53 F | English | White Irish | Phone |
| 04.2 | Pre-diabetes | Anxiety, agoraphobia, pain | Several LW visits and referrals | In-house | 43 F | English | Bangladeshi | Phone |
| 04.3 | T2D | Lifestyle, bereavement, employment. | Several LW visits and referrals | In-house | 35 F | English | Bangladeshi | Online |
| 04.4 | Pre-diabetes | Depressed mood, stress. | Several LW visits and referrals | In-house | 61 F | English | White British | Phone |
| 04.5 | Pre-diabetes | Housing, immigration status. | 1 LW visit | Centrally managed | 38 M | Bengali | Bangladeshi | Phone, advocate |
| 04.6 | GDM,  QDS > 20 | Housing, depressed mood, stress | 2 LW visits and 1 referral | Centrally managed | 36 F | English | Bangladeshi | Phone |
| 04.7 | QDS > 20 | Depressed mood, stress. | Several LW visits and referrals | In-house | 49 M | English | Black British | Online |
| 04.8 | GDM,  QDS> 20 | Housing. | 1 LW visit and 1 referral | Centrally managed | 27 F | English | Bangladeshi | Phone |

LW link worker; F female; M male; QDS Q-Diabetes Score, GDM: Gestational Diabetes Mellitus

**Table S3.** Characteristics of link workers interviewed

| **Code** | **Type** | **Gender** | **Age** | **Time worked in TH** | **Ethnicity** | **Interview mode** |
| --- | --- | --- | --- | --- | --- | --- |
| 02.1 | In-house | Female | 20-30 | <1 year | White | online |
| 02.2 | In-house | Female | 30-40 | >5 years | White | phone |
| 02.3 | DES funded | Male | 40-50 | <1 year | South Asian | online |
| 02.4 | CCG funded | Male | 40-50 | >5 years | South Asian | online |
| 02.5 | CCG funded | Female | 40-50 | >5 years | White | online |
| 02.6 | CCG funded | Female | 50-60 | >5 years | White | online |
| 02.7 | DES funded | Female | 40-50 | <1 year | South Asian | online |
| 02.8 | DES funded | Female | 40-50 | >5 years | South Asian | online |
| 02.9 | DES funded | Female | 30-40 | 1 to 5 years | White | online |
| 02.10 | In-house | Female | 30-40 | <1 year | White | online |
| 02.11 | CCG funded | Female | 40-50 | >5 years | South Asian | online |

DES Direct Enhanced Scheme; CCG Clinical commissioning Group; TH Tower Hamlets

**Table S4.** Characteristics of primary care clinicians interviewed

| **Code** | **Role** | **Contract** | **Gender** | **Age** | **Time worked in TH** | **Ethnicity** | **Interview mode** |
| --- | --- | --- | --- | --- | --- | --- | --- |
| 01.1 | GP | Partner | Male | 40-50 | >5 years | White | online |
| 01.2 | GP | Locum | Male | 30-40 | 1-5years | Asian | phone |
| 01.3 | GP | Salaried | Female | 30-40 | >5 years | Asian British | online |
| 01.4 | Physiotherapist | Salaried | Female | 30-40 | <1 year | White | online |
| 01.5 | GP | Partner | Male | 40-50 | >5 years | White | online |
| 01.6 | GP | Partner | Female | 30-40 | >5 years | Asian British | online |
| 01.7 | GP | Partner | Female | 40-50 | >5 years | Asian British | online |
| 01.8 | Nurse | Salaried | Female | 40-50 | >5 years | White | online |
| 01.9 | HCA | Salaried | Female | 40-50 | >5 years | Asian British | online |
| 01.10 | Nurse | Salaried | Female | 40-50 | >5 years | White | online |
| 01.11 | Nurse | Salaried | Female | 40-50 | >5 years | Black | online |

HCA Health care Assistant; TH Tower Hamlets

**Table S5.** Characteristics of VCS members interviewed

| **Code** | **Gender** | **Age** | **Role** | **Activity type** | **Funding** | **Interview mode** |
| --- | --- | --- | --- | --- | --- | --- |
| 03.1 | Female | 30-40 | Welfare advisor | Welfare advice, physical activity, cultural activities | Methodist Church, National Lottery | online |
| 03.2 | Female | 40-50 | Coordinator | Welfare advice, community kitchen, day centre, walking group | CCG and TH Council | online |
|  | Female | 40-50 | Coordinator |  |  |  |
| 03.3 | Female | 30-40 | Employment advisor | Employability and Skills | Donations, legacies, public contracts, grants | online |
| 03.4 | Male | 40-50 | Social Welfare advisor | Welfare advice | Donations, legacies, public contracts, grants | online |
| 03.5 | Female | >70 | Founder | Day centre, community kitchen, physical activity | Donations, public contracts | online |
| 03.6 | Female | 50-60 | CEO | Mental Health Support | Donations, public contracts | Online |
|  | Female | 40-50 | Operational director |  |  |  |
| 03.7 | Female | 40-50 | Legal/welfare advisor | General and specialised (DV) legal and welfare advice | Corporate funding, donations, lottery, public contracts | online |
| 03.8 | Male | 50-60 | Project Manager | Physical activity programme | Donations, legacies, public contracts, grants | online |
| 03.9 | Female | 40-50 | CEO | Weight management, healthy lifestyle and physical activity programme | Donations, legacies, public contracts, grants | online |
|  | Female | 40-50 | Project Manager |  |  |  |
| 03.10 | Male | 40-50 | Community navigator | Support and signposting | Public Health England | online |
| 03.11 | Male | 40-50 | Coordinator | Day centre, physical activity – special focus on patients with mental health conditions | Local council | online |
| 03.12 | Female | 40-50 | Provider | Food Bank | Donations, legacies, public contracts, grants | online |
|  | Female | 40-50 | CEO |  |  |  |
|  | Female | 20-30 | Volunteer |  |  |  |
| 03.13 | Male | 60-70 | Provider | Physical Activity programme | Donations, legacies, public contracts, grants | in person |

CCG Clinical commissioning Group; TH Tower Hamlets; DV Domestic Violence

**Box S5.** Characteristics of VCS and NDPP activities observed

| **VCS1 – community-based health promotion programme** |
| --- |
| - Consisted of a holistic weight management programme covering nutritional and lifestyle topics over 12 weeks, as well as a social, peer-led 8-week course about different aspects of good health and wellbeing for people with long term conditions (including T2D, high blood pressure, cardiovascular disease, chronic kidney disease). - Held in the organisation’s main office. Free for attendees and commissioned by the local authority and CCG. - Observed for a total of 8 hours over a period of 2 months between September and November 2021. |
| **VCS2- community-based physical activity and wellbeing programme** |
| - Consisted of a physical activity and wellbeing programme aimed at young people aged 16 to 26. The programme was run by a wellbeing coach and combined classroom-based interactive sessions on ’healthy’ lifestyle with practical physical activity sessions in open-access exercise facilities. - Held in a local community centre. Free for attendees and funded by a 3-year grant support line from the central government. - Observed over a period of 2 weeks between August and September 2021 (total 3 visits, 12 hours). |
| **NDPP sessions** |
| - Consisted of group sessions focusing on weight management, physical activity and healthy diet, delivered by health and wellbeing coaches fortnightly and then monthly over a period of 9 months. Available to those patients registered with a GP practice in the local area and diagnosed as being at high risk of developing T2D. - Held online. Free for attendees, delivered by a private provider and publicly commissioned by Northeast London Health and Care Partnership. - Observed over a period of 2 months from September to November 2021 (total 4 visits, 6 hours). |
| **VCS meetings** |
| - Consisted of monthly meetings with local VCS organisations, link workers and community navigators aimed at showcasing available activities and resources, providing relevant updates, and creating opportunities for collaborative partnership across organisations - Held online during Covid 19 and in person after September 2021. Open to the public and self-organised/funded - Observed almost monthly between March 2021 and April 2022 (total 10 visits, 10 hours). |
