# Supplementary material for: Unravelling the potential of social prescribing in individual-level type 2 diabetes prevention: a mixed-methods realist evaluation
Source: BMC Med. 2023 Mar 13;21:91. doi: 10.1186/s12916-023-02796-9 (PMC10008720; doi:10.1186/s12916-023-02796-9)
Supplement: Supplementary file 3 — Additional file 3. COMCs developed in the realist mixed-methods evaluation. Figure S1. Realist evaluation COMCs. [file 12916_2023_2796_MOESM3_ESM.docx]

**Additional File 3. COMCs developed in the realist mixed-methods evaluation**

Drawing on quantitative data and interviews with patients and service providers (including comparisons with NDPP where possible and relevant), we developed four CMOCs that explain why (and how) accessible, holistic, sustained and integrated SP approaches were key for contributing to T2D prevention in communities at high risk. These four CMOCs are synthesised in Figure 3 and explained in more detail below and in Figure S1:

*CMOC1: In a context where services relevant to T2D prevention (namely, NDPP) are hard to reach by those in need (characterised by lower referral rates to NDPP, especially amongst those of lower socioeconomic status and with co/multimorbidity) (C), accessible approaches (characterised by broad eligibility criteria, proactive and welcoming approaches) (M) proved key for ensuring enhanced service uptake(O).*

*CMOC2: In a context of great social vulnerability (characterised by greater socioeconomic deprivation and ethnic/racial minoritised status amongst high-risk patients referred into SP) (C), holistic approaches (characterised by broad conversations prior to and during referrals and wide service remit) (M) proved key for ensuring personalised and contextually sensitive care (O).*

*CMOC3: In a context of ongoing and fluctuating care needs (especially insofar as underlying drivers persisted) (C), sustained approaches (characterised by ongoing and open-ended services and continuity of care with named service providers) (M) proved key for the development of therapeutic relationships over time (O).*

*CMOC4: In a context of great clinical care need (characterised by high rates of co/multimorbidity amongst high-risk patients referred into SP) (C), integrated approaches (characterised by locally embedded, well-coordinated primary care and VCS) (M) proved key for ensuring enhanced service accountability and responsiveness (O).*

**Figure S1. Realist evaluation COMCs**

**
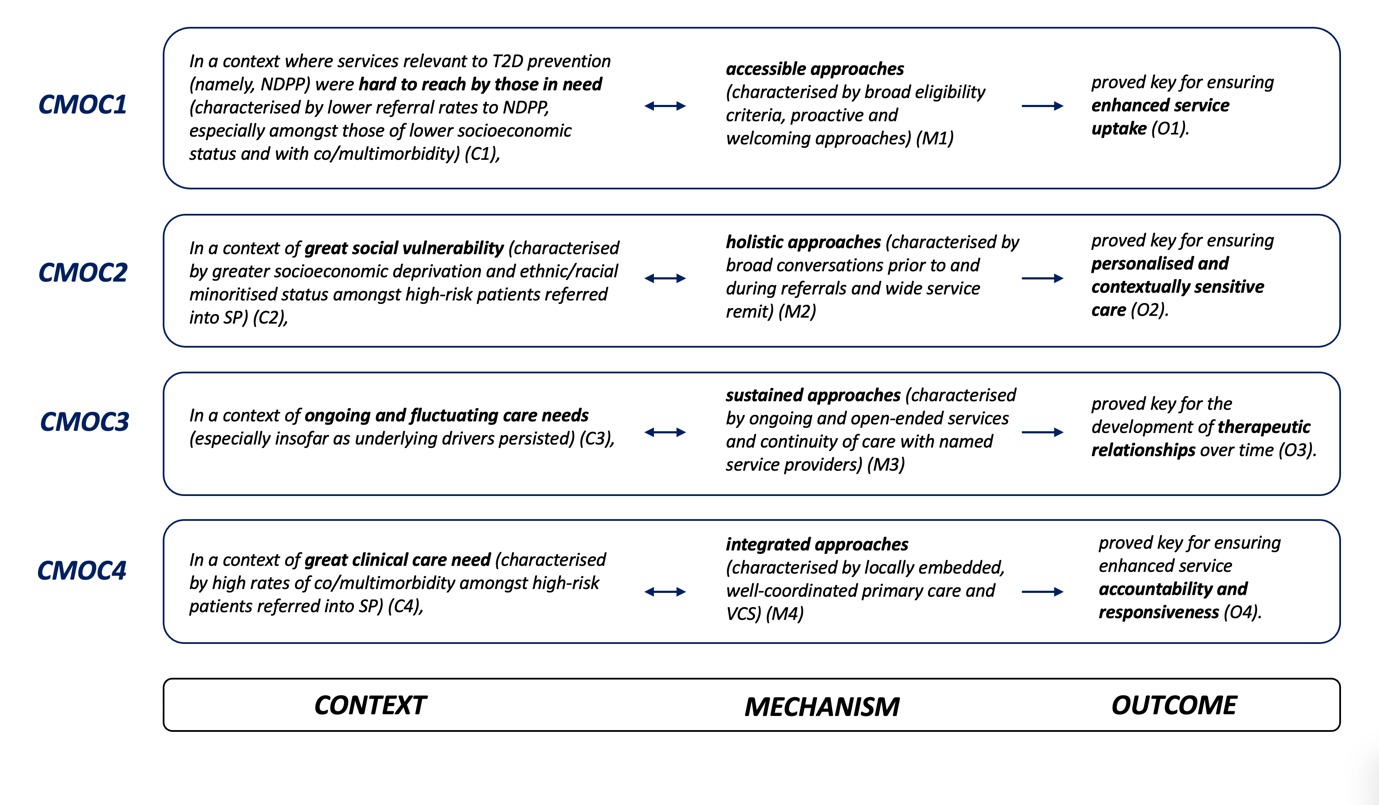
**
